# Tracking Gene Expression of Single Mitochondria in Live Neurons Using Nanotweezers

**DOI:** 10.1021/jacs.6c02802

**Published:** 2026-06-09

**Authors:** Annie Sahota, Binoy Paulose Nadappuram, Siân C. Allerton, Flavie Lesept, Jack H. Howden, Suzanne Claxton, Yaxian Liu, Francesco A. Aprile, Josef T. Kittler, Michael J. Devine, Aleksandar P. Ivanov, Joshua B. Edel

**Affiliations:** † Department of Chemistry, Molecular Science Research Hub, 4615 Imperial College London, London W12 0BZ, U.K.; ‡ Institute of Chemical Biology, Molecular Science Research Hub, Imperial College London, London W12 0BZ, U.K.; § Department of Pure and Applied Chemistry, University of Strathclyde, Glasgow Gl 1XL, U.K.; ∥ Department of Neuroscience, Physiology and Pharmacology, 4919 University College London, Gower Street, London WC1E 6BT, U.K.; ⊥ Kinases and Brain Development Lab, 376570 The Francis Crick Institute, 1 Midland Road, London NW1 1AT, U.K.; # Mitochondrial Neurobiology Lab, 376570 The Francis Crick Institute, 1 Midland Road, London NW1 1AT, U.K.; 7 Department of Clinical and Movement Neurosciences, UCL Queen Square Institute of Neurology, University College London, London WC1N 3BG, United Kingdom

## Abstract

Neurons are highly polarized cells that depend on mitochondria for energy and signaling homeostasis. Importantly, energy and signaling requirements vary considerably across individual neurons both spatially and temporally. Therefore, to fully understand neuronal mitochondria, methods are needed to analyze mitochondria in live cells over time. The nanotweezer, a minimally invasive single-cell sampling technique, enables precise extraction of individual mitochondria from defined subcellular locations. Here, we combine single-mitochondrial extraction from live neurons with targeted mitochondrial gene expression tracking and mtDNA profiling to develop a platform for live-cell single-mitochondrion tracking and analysis. By tracking the expression of specific mitochondrially encoded genes in the same neurons over time, we reveal preliminary data showing a downregulation of mitochondrial genes MT-ND1 and MT-ATP6 following exposure to α-synuclein aggregates, independent of the proximity of the aggregates to the sampled mitochondria. Our approach provides a proof-of-concept for precise, temporal measurements of mitochondrial composition and targeted gene expression *in vitro* at single-organelle resolution, opening opportunities for single-cell and single-organelle studies of neuronal mitochondrial heterogeneity and its perturbation in models of neurodegeneration.

## Graphical abstract

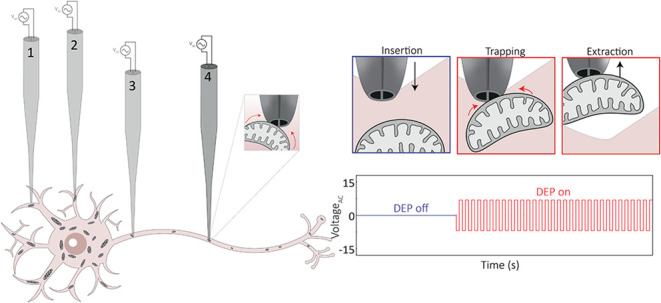

## Introduction

Neurons rely heavily on mitochondria because of their disproportionately high ATP demand compared to other cell types.^1^ Mitochondrial dysfunction is a core feature of aging and neurodegeneration, including Parkinson’s disease (PD),^2^ Alzheimer’s disease (AD),^3^ and amyotrophic lateral sclerosis (ALS).^4^ Decreased mitochondrial DNA (mtDNA) copy number and elevated mtDNA heteroplasmy have been identified in neurodegenerative diseases.^5^ Dysregulation of mitochondrial gene expression and mitochondrial dynamics has also been observed, affecting synaptic transmission, membrane potential maintenance, calcium buffering, and apoptosis.^6^

Mitochondrial subpopulations are evident within the neuronal subcompartments.^7^ For example, synaptic mitochondria are distinct in their morphology,^8,9^ proteomic profiles,^10^ enzyme activity,^11^ calcium buffering,^12^ and vulnerability compared to nonsynaptic mitochondria.^13^ This, combined with the knowledge that specific compartments of a neuron may be selectively degenerated in neurodegenerative diseases before the loss of the neuron,^14,15^ highlights the need to study the heterogeneity of compartmentalized mitochondria within single neurons. Single-cell analysis technologies have enabled the characterization of mitochondrial heteroplasmy in neurons and hold great potential for a better understanding of heteroplasmy at the single-cell level.^16,17^ For example, mitochondrial transplantation has been performed using FluidFM, in which multiple mitochondria and portions of the mitochondrial network were transplanted from HeLa cells into U2OS cells and primary human endothelial keratinocytes.^18^ However, single-cell sampling techniques based on micropipettes or nanopipettes require mechanical sampling or aspiration of cytoplasmic fluid to extract cellular contents. More recently, an automated nanobiopsy platform has been developed for mitochondrial transplantation and sensing.^19^

Understanding the spatial differences in mitochondrial distribution in neurons and the localized effects of pathological markers, such as protein aggregation, on neuronal mitochondria could be further advanced by analyzing the composition of individual mitochondria in live neurons. This could enable precise mitochondrial mechanisms to be unraveled and novel treatment targets to be identified at single-organelle resolution. Steps toward this have been shown in a recent high-resolution imaging study, which observed mitochondrial translation products in the axons, dendrites, and synaptic regions of hippocampal neurons.^20^ Additionally, single-cell sampling of individual mitochondria from neurons using micropipettes and nanopipettes has been combined with sequencing to study mitochondrial heteroplasmy.^21–23^ However, no method currently enables the isolation and analysis of individual mitochondria for tracking the mitochondrial gene expression in live cells. Further advances in single-cell sampling approaches and analysis methods for examining the composition of individual mitochondria in neurons have the potential to substantially improve our understanding of basic mitochondrial biology and to facilitate the discovery of novel cellular mechanisms and the development of improved therapeutic interventions for neurodegenerative diseases.^24^

To bridge this gap, we build on our nanotweezer technology, which enables minimally invasive biomolecular extraction from living cells via localized dielectrophoretic (DEP) trapping rather than bulk cytoplasmic aspiration. We previously demonstrated single-mitochondrion extraction from neuronal axons and repeated cytoplasmic sampling without compromising viability.^25–27^ However, dynamic sampling of mitochondria from single cells or the integration of mitochondrion extraction with the quantification of mitochondrially encoded transcripts was not performed in this work. More recently, we have shown that multiple cytoplasmic extractions can be performed on the same live neuron, including from neurites, without affecting cell viability, a feat particularly notable given the delicate nature of neurons *in vitro*.^27^ Here, we develop an approach that combines multiple individual mitochondrial extractions from the same neuron with single-mitochondrial mtDNA and mtRNA analysis, enabling single organelle profiling in live neurons. Furthermore, we show that individual mitochondria can be isolated from specific neuronal locations and at different time points within the same cell following uptake of α-synuclein (α-syn) oligomers, thereby enabling the tracking of a selected set of mitochondrially encoded transcripts in live neurons in an acute toxicity model.

## Results and Discussion

### Extraction of Single Mitochondria from Live Neurons

To isolate individual mitochondria from live neurons, mitochondria in neurites were targeted for nanobiopsy extraction using the nanotweezer (Figure 1a). Mitochondria were fluorescently labeled *in vitro* by transfecting neurons with mtDsRed (Figure 1b), which enabled specific targeting of a mitochondrion’s matrix. Using bright-field imaging (Figure 1b, inset), the nanotweezer was manually inserted into a neuron adjacent to a labeled mitochondrion. The extraction of a single mitochondrion was then monitored by fluorescence following nanotweezer insertion into the cell (Figure 1c­(i), Figure 1d­(i), Figure 1e­(i)). An AC voltage was applied adjacent to a mitochondrion, which enabled the mitochondrion to become trapped at the nanotweezer tip by dielectrophoresis (DEP) (Figure 1c­(ii), Figure 1d­(ii), Figure 1e­(ii)), as illustrated by a shift in mitochondrial fluorescence closer to the nanotweezer tip. The nanotweezer was then removed from the cell, extracting the trapped mitochondrion, as illustrated by a loss of fluorescence derived from the isolated mitochondrion (Figure 1c­(iii), Figure 1d­(iii), Figure 1e­(iii)). To further confirm successful mitochondrial extraction from the cell, fluorescence was observed on the nanotweezer tip outside the cell (Figure 1f), which persisted when moving the nanotweezer, confirming that the mitochondrion was trapped on the nanotweezer tip.

**Figure 1:**
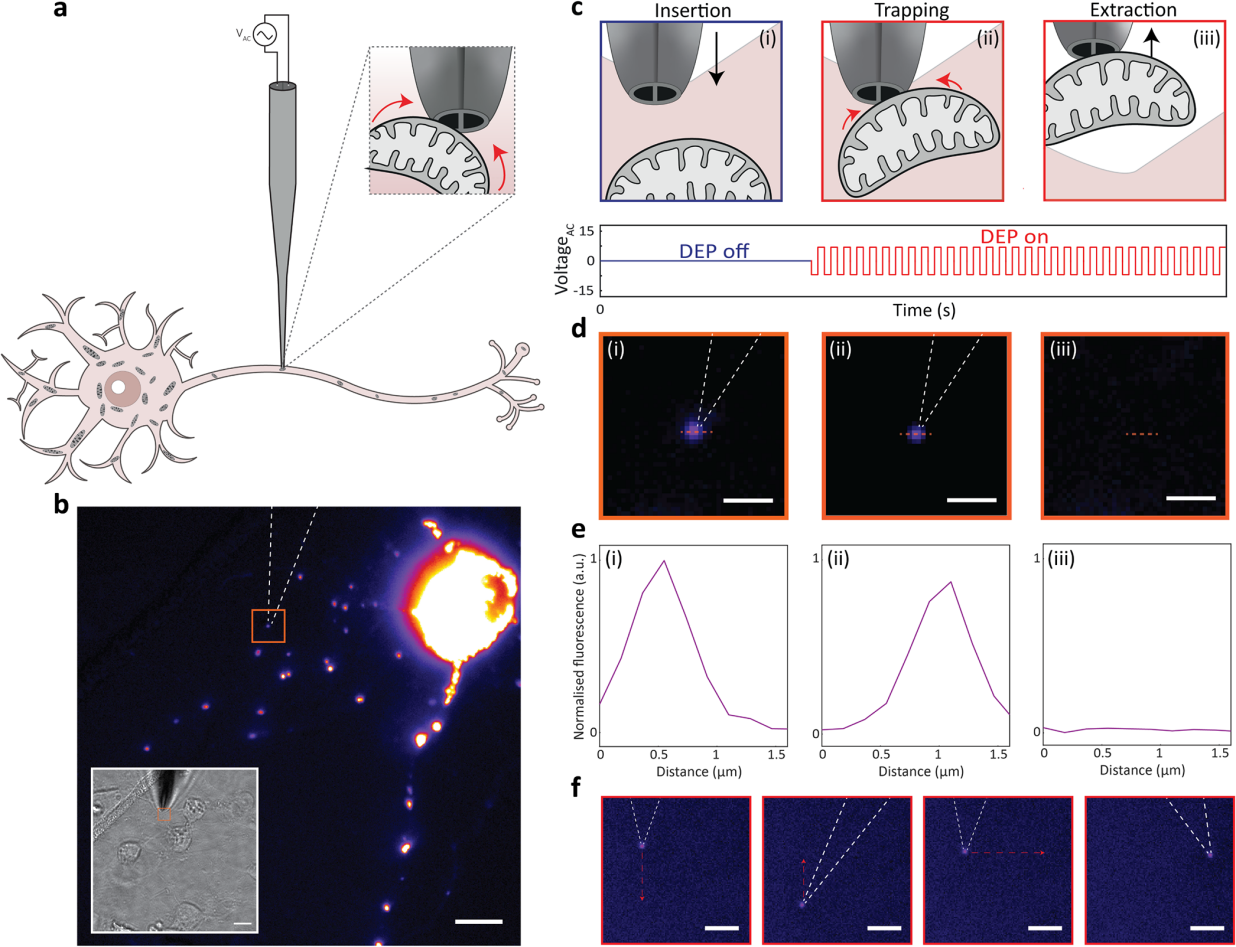
Single mitochondrion isolation. a) Schematic illustrating the trapping of a single mitochondrion from a neuron using the nanotweezer. b) Fluorescent image of an mtDsRed-labeled neuron and the corresponding bright-field image (inset) of the nanotweezer inserted into the cell adjacent to a single mitochondrion within the cell. The orange box represents the region containing the targeted mitochondrion. The dashed white lines in the fluorescence image represent the position of the nanotweezer. Scale bars = 10 μm. c) Schematic showing insertion of the nanotweezer into the cell adjacent to a mitochondrion (i), DEP trapping of the mitochondrion via application of an AC voltage (ii), and extraction of the mitochondrion via removal of the nanotweezer from the cell (iii). d) Corresponding fluorescent images of the targeted mitochondrion after insertion of the nanotweezer (i), DEP trapping at the nanotweezer tip (ii), and removal of the nanotweezer from the cell (iii), showing the complete loss of the targeted mitochondrion’s fluorescence. Scale bars = 2 μm. e) Corresponding fluorescence traces of the targeted mitochondrion measured over the distance shown by the dashed line in (d), showing levels of mitochondrial fluorescence before application of DEP (i), trapping of the mitochondrion at the tip upon application of DEP (ii), and loss of mitochondrial fluorescence following the removal of the trapped mitochondrion (iii). f) Fluorescent images of the nanotweezer tip outside the cell after the removal of the trapped mitochondrion, where the nanotweezer was moved in *x* and *y* directions, and the fluorescent mitochondrion moved simultaneously, confirming the presence of a trapped mitochondrion at the tip. Scale bars = 5 μm.

To assess the preservation of the mitochondrial membrane potential (MMP) of the mitochondrion trapped using the nanotweezer and to confirm that intact mitochondria were isolated from cells, a single mitochondrial nanobiopsy was performed with mitochondria labeled with tetramethylrhodamine methyl ester (TMRM), a fluorescent probe readily sequestered by mitochondria with intact and active MMP (Figure S1). The fluorescence of the mitochondrion was observed before and during nanotweezer DEP application, as well as after mitochondrial extraction from the cell, where the nanotweezer was maintained in the cell’s culture medium (Figure S1a). Mitochondrial extraction was confirmed as before, where an increase in fluorescence was observed at the nanotweezer tip upon application of DEP, showing that the targeted mitochondrion moved toward the nanotweezer tip (Figure S1b). The preservation of MMP of isolated mitochondria was also validated, with no loss of mitochondrial fluorescence observed during the trapping procedure, and fluorescence remaining on the nanotweezer tip after extraction from the cell (Figure S1c). To investigate possible effects on the mitochondria that remained in the cell following nanobiopsy, the fluorescence of neighboring mitochondria and non-neighboring mitochondria in the same cell was measured during the nanobiopsy procedure. This was compared to the fluorescence of mitochondria in neighboring cells that were not subjected to nanobiopsy. As illustrated by Figure S1d, the fluorescence intensity of the mitochondria remained constant, showing that there were minimal effects on the mitochondrial membrane potential of mitochondria in the same cell. The long-term effects of extracted mitochondria on MMP were investigated by measuring the fluorescence intensity of the trapped mitochondria on the nanotweezer tip outside the cell, demonstrating that mitochondria isolated using the nanotweezer retained MMP for at least 90 min.

### Single Mitochondrion Analysis

Having demonstrated that intact mitochondria can be extracted from neurons using the nanotweezer without affecting MMP, protocols to analyze the content of a single mitochondrion were developed. First, mtDNA was successfully detected using qPCR following single mitochondrial lysis and purification (Figure 2a). mtDNA was detected exclusively in nanobiopsy samples, as evidenced by the absence of amplification in qPCR-negative and purification controls. The negative control was a no-template control that included all qPCR reagents, along with nuclease-free water in place of DNA. The purification control comprised the lysis and purification steps applied to nuclease-free water. To investigate potential nonspecific adherence of mitochondria or mtDNA to the nanotweezer, the nanotweezer was inserted into the cell’s media and into the cell itself without the application of DEP. These additional controls showed minimal amplification compared with that of extracted mitochondria. Following the detection of mtDNA from each mitochondrion, mtDNA copy numbers from individual mitochondria were obtained using absolute quantification (Figure 2b, Figure S2). Amplification was observed in 9 of 14 mitochondria extracted using the nanotweezer, with copy numbers ranging from 0 to 13 per mitochondrion. These copy numbers are similar to the previously reported estimates of mtDNA copies per mitochondrion.^28,29^ It is important to highlight that absolute qPCR quantification is based on the construction of a standard curve where, despite the standard curve constructed in this work having a high *R* ^2^ value (0.99) and the primers having good PCR efficiency (96%), slight variations from pipetting, amplification efficiencies per well, and sample mixing may affect the quantification, particularly when assessing copy numbers in the range described here. Single mitochondrial mtDNA quantification used in this manner may therefore be suited for relative comparisons between samples processed in parallel rather than for examining absolute numbers within a single sample type. For example, the method described here could be used to compare mtDNA copy numbers within subcellular compartments of the same neuron, as recently described.^30^ Alternatively, the method could be used to compare mitochondria in healthy and PD neurons.

**Figure 2:**
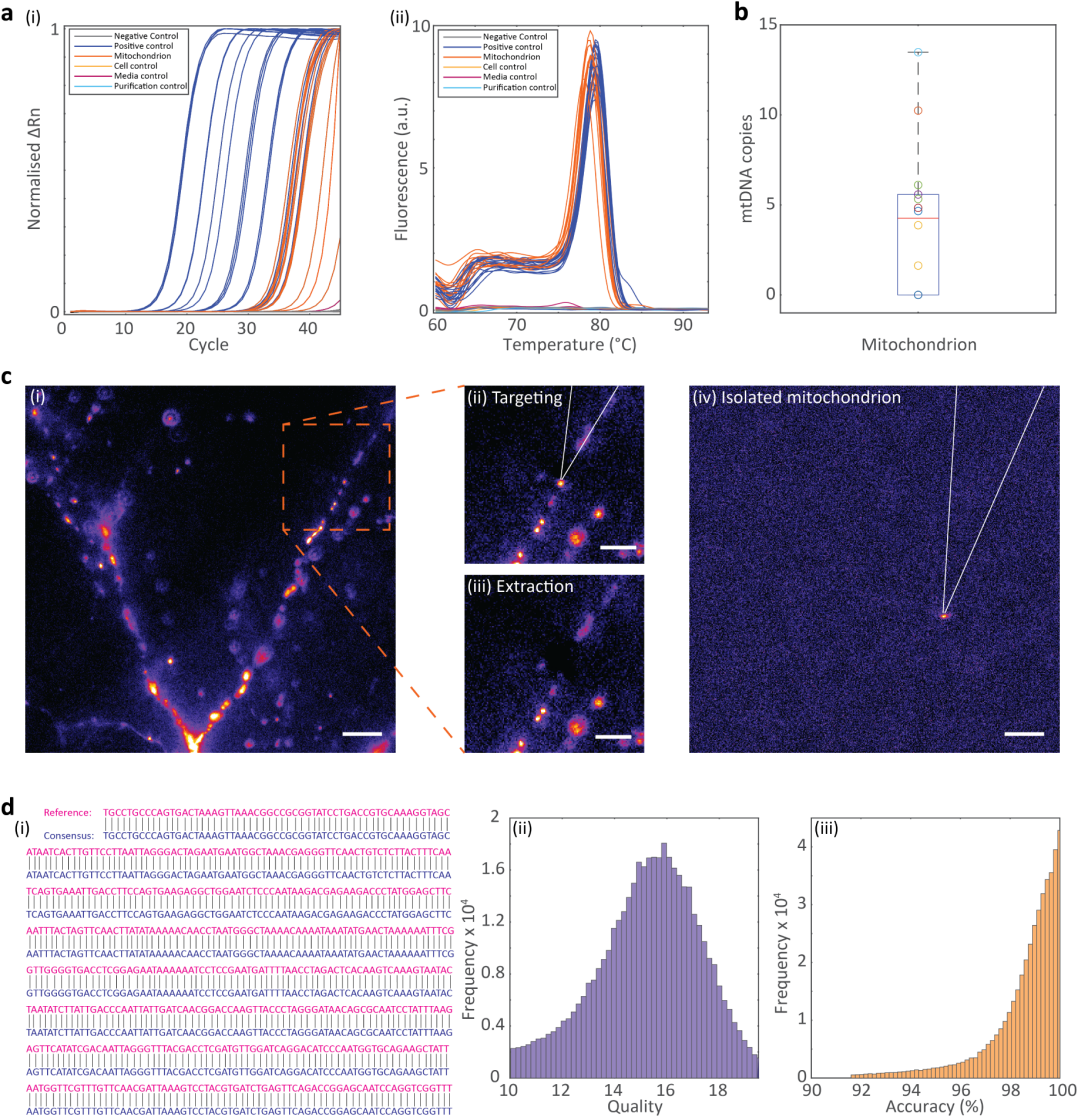
Single mitochondrion mitochondrial DNA analysis. a) qPCR amplification plot (i) and melt curve (ii) of mtDNA isolated from individual mitochondria extracted from neurons compared to controls. qPCR was performed using primers for MT-CO1. b) mtDNA copy numbers calculated for each mitochondrion (*n* = 14 mitochondria). mtDNA copy numbers were calculated from a standard curve constructed from the positive control of a dilution series of known amounts of DNA that comprised the target amplicon. c) Fluorescent images of mtDsRed-transfected neurons and targeted mitochondrion for extraction and target amplicon sequencing. The dashed box in (i) represents the targeted region in the cell. The nanotweezer was targeted to an individual mitochondrion in this region (ii), followed by the extraction of the mitochondrion (iii). Isolation of the mitochondrion was confirmed by a loss of fluorescence in the cell following extraction and the detection of fluorescence at the nanotweezer tip outside the cell (iv). The white lines represent the nanotweezer. (i) and (iv) Scale bars = 10 μm. (ii) and (iii) Scale bars = 5 μm. Summary statistics for boxplots: center = median; bounds of box = IQR 25th and 75th percentile; whiskers = minimum and maximum within 1.5 IQR. d) Overall alignment (i), quality distribution (ii), and accuracy distribution (iii) of the reads from nanopore sequencing of a 558 bp region of the mtDNA from the extracted mitochondrion.

Mitochondria extracted using the nanotweezer were then analyzed by DNA nanopore sequencing. Single-mitochondrial sequencing without amplification was unsuccessful, likely due to the small number of mtDNA copies per mitochondrion and the potential loss of material during library preparation. To address this, mitochondrial DNA was sequenced from PCR-amplified samples of a 558 bp region of the MT-RNR2 gene. A single mitochondrion was isolated from a neuron using the nanotweezer (Figure 2c), lysed, and purified, and then a 558 bp region within the MT-RNR2 gene was amplified using a high-fidelity polymerase. PCR-amplified material was then successfully sequenced by nanopore sequencing, as demonstrated by 100% alignment of the consensus sequence with the reference sequence (Figure 2d­(i)), a median quality score of 15.0 (Figure 2d­(ii)), and a median accuracy score of 98.4% (Figure 2d­(iii)).

A protocol for analyzing RNA from a single mitochondrion was also developed. Because the amount of mtDNA per mitochondrion can vary, mtDNA must be excluded from gene expression analysis. Because of mitochondrially encoded RNAs not containing introns, this could not be performed using primers that spanned exon–exon junctions in RT-qPCR. To address this, the purification step following mitochondrial lysis was modified by utilizing surface-functionalized magnetic beads with target-specific binding regions. The target-specific binding regions comprised oligonucleotides with a 25-nucleotide repeat of thymine nucleotides for binding to polyadenylated RNAs and oligonucleotides with sequences complementary to 12S rRNA. This enabled the selective enrichment of mtRNAs during purification and avoided the carryover of mtDNA, which may otherwise lead to a false-positive gene expression analysis. The single mitochondrion mtRNA analysis method is summarized in Figure 3a.

**Figure 3:**
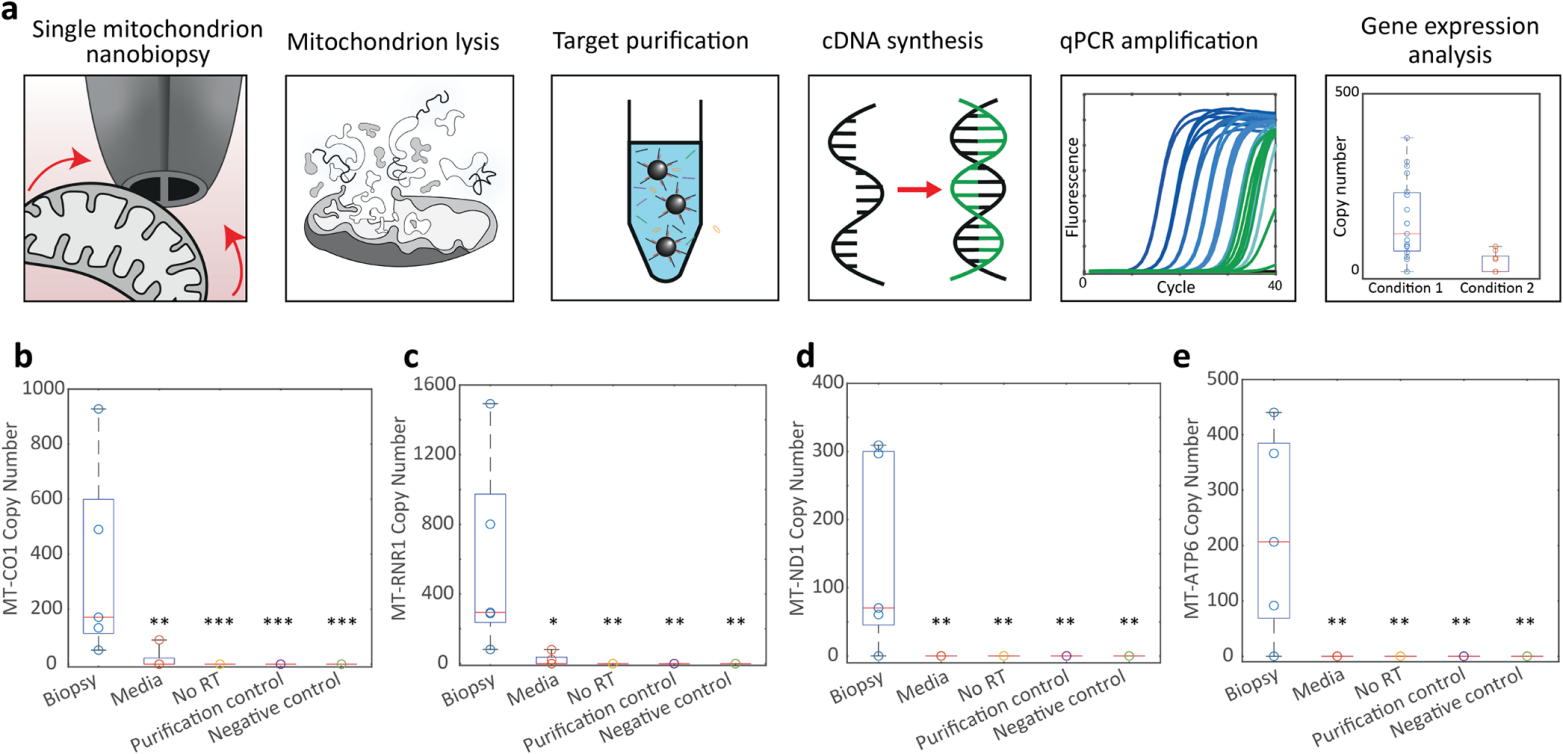
Single mitochondrial RNA analysis. a) Schematic of the single mitochondrial mtRNA analysis procedure. b) RT-qPCR copy numbers derived from single mitochondrial nanobiopsies, cell culture media controls, no RT controls, and RT-qPCR negative controls for MT-CO1, c) MT-RNR1, d) MT-ND1, and e) MT-ATP6 expression (*n* = 5). Statistical analysis was performed using the Kruskal–Wallis nonparametric test between biopsies and all controls (*P < 0.05, **P < 0.01, ***P < 0.001, ^n.s^ *P* = not significant). Summary statistics for boxplots: center = median; bounds of box = IQR 25th and 75th percentile; whiskers = minimum and maximum within 1.5 IQR.

The isolation and detection of mtRNA in the absence of mtDNA were successful, as evidenced by the absence of mtDNA in the no-RT control. The detection of mtRNA from the nanobiopsy extractions was further supported by the detection of a significantly higher mtRNA concentration in mitochondria isolated by nanobiopsy than in the purification, media, and RT-qPCR controls. The purification control served as an additional negative control, in which water was analyzed using the method shown in Figure 3a to ensure that the detected mtRNA did not arise from contamination during the analysis. The medium control represented mtRNA derived from nanobiopsy from media outside the cell, accounting for any potential cell-free mitochondria or mtRNA. Combined, these results show that mtRNA can be analyzed from an individual mitochondrion extracted using the nanotweezer and that the detected mtRNA is derived from the extracted mitochondrion. However, low-template PCR-derived absolute copy numbers should be interpreted cautiously beyond this proof-of-concept.

### Spatially Resolved Single Mitochondrial Extraction Shows That the Proximity of α-Synuclein Oligomers Does Not Determine Mitochondrial Gene Expression of Specific Transcripts

To demonstrate the capability to probe mitochondrial gene expression profiles of specific mitochondrially encoded genes within a single neuron, the interaction between N-terminally acetylated α-synuclein (hereinafter referred to as α-syn) aggregates and a selected set of mitochondrial transcripts was investigated in an acute toxicity model. Previous work has suggested a direct interaction between α-syn aggregates and neuronal mitochondria,^31–33^ in which α-syn (including oligomeric species) can pass through cellular membranes and localize within mitochondrial membranes near mitochondrial proteins, such as ATP synthase (complex V) and mitochondrial complex I.^34–37^

As small, soluble oligomers are considered the most toxic form of α-syn,^38^ an aggregation mixture containing oligomers was prepared. Aggregation and oligomer formation were confirmed by atomic force microscopy (AFM), as evidenced by an increase in equivalent disc radius and a broader size distribution with incubation (Figure S3). To investigate the effect of α-syn oligomers only, the protein mixtures were labeled with an α-syn aggregate-specific fluorescently labeled aptamer.^39^ The fluorescently labeled α-syn oligomers were incubated with mtDsRed-labeled neurons for 24 h to allow uptake into cells and then washed to remove excess (Figure 4a). This enabled imaging of both α-syn oligomers and mitochondria in the neurons.

**Figure 4:**
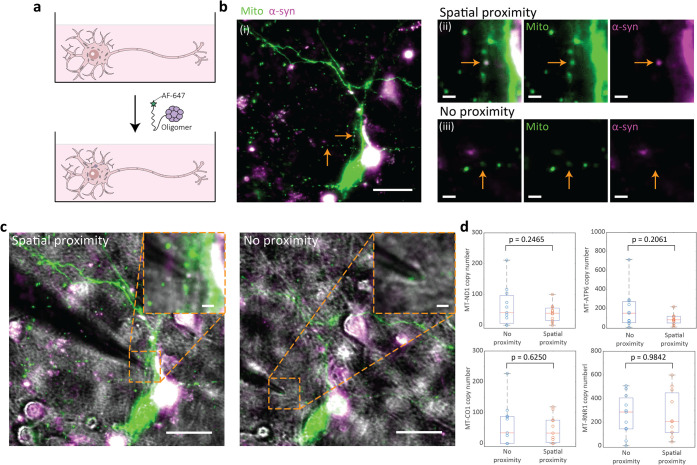
Expression of specific mitochondrially encoded genes from single mitochondrion biopsies in spatial proximity with α-synuclein oligomers. a) Schematic of the application of fluorescently labeled α-synuclein oligomers to neurons. b) Fluorescent images of mtDsRed-labeled DIV 14 neurons following 24 h incubation with fluorescently labeled α-synuclein oligomers (i). Some of the fluorescently labeled α-synuclein oligomers were in spatial proximity to the mitochondria in the cell (ii), and some were not (iii). Mitochondria are labeled in green, and α-synuclein oligomers are labeled in magenta. (i) Scale bar = 20 μm, (ii) and (iii) scale bars = 2 μm. c) Fluorescent images of mtDsRed-labeled neurons with fluorescently labeled α-synuclein oligomers overlaid with bright-field images of nanobiopsies of single mitochondria in spatial proximity with α-synuclein oligomers and in no proximity with α-synuclein oligomers. Scale bars = 20 μm. Inset scale bars = 2 μm. d) Boxplots of MT-ND1, MT-ATP6, MT-CO1, and MT-RNR1 copy numbers derived from individual biopsies of mitochondria proximal and not proximal to α-synuclein oligomers (*n* = 11 mitochondria). Statistical analysis was performed using a Wilcoxon test (MT-ATP6, MT-CO1) or a paired *t*-test (MT-ND1, MT-RNR1). Summary statistics for boxplots: center = median; bounds of box = IQR 25th and 75th percentile; whiskers = minimum and maximum within 1.5 IQR.

As shown by the overlap between the α-syn channel and the mitochondrial channel in Figure 4b­(i), some of the α-syn oligomers were near individual mitochondria (Figure 4b­(ii)), and some were not (Figure 4b­(iii)) following the uptake of the α-syn oligomers in the cells. Spatial proximity was defined by the overlap between α-syn and mitochondrial channels, as shown in Figure 4b­(ii). To investigate whether this proximity influenced single mitochondrial gene expression of specific mitochondrial transcripts, individual mitochondria overlapping and not overlapping with α-syn oligomers were extracted using the nanotweezer (Figure 4c). The nanotweezer’s high spatial precision enabled the specific selection of mitochondria. Gene expression of specific transcripts from each mitochondrion was analyzed using the method outlined in Figure 3a using standard curves for absolute copy number quantification of each mitochondrion (Figure S4). To minimize technical errors in the absolute quantification of single organelles, mitochondrial transcripts were analyzed in parallel alongside negative controls within the same qPCR plate, where possible, to enable relative comparisons and control for technical variability.

Surprisingly, no significant difference between the expression of MT-ND1, MT-ATP6, MT-CO1, and MT-RNR1 was observed in mitochondria that overlapped with α-syn oligomers and mitochondria that did not. Therefore, these findings do not support a direct influence of α-syn oligomers on these mitochondrially encoded genes.

### Single-Cell Targeted Mitochondrial Gene Expression Tracking Shows Suppression of MT-ND1 and MT-ATP6 Due to α-Syn Oligomer Exposure

To investigate the effects of α-syn oligomers on the expression of a specific mitochondrial gene panel in single neurons, the nanotweezer was used to extract multiple individual mitochondria from the same live neuron over 24 h in an acute α-syn toxicity model. Single-cell gene expression tracking would enable the same live cell to be probed, uncovering changes in gene expression and unmasking the heterogeneity of cell populations that is missed in bulk studies. With this aim in mind, the nanotweezer was used to initially examine the potential effects of α-syn oligomers on the expression of specific mitochondrial transcripts at the single-neuron level.

Figure 5a shows a schematic of the single mitochondrial extraction procedure performed in the same cells using the nanotweezer to track specific mitochondrial expression after the addition of α-syn oligomers. Using gridded cell culture dishes to locate the regions of neurons undergoing nanobiopsy, multiple single mitochondria were extracted from the same cell under normal culture conditions (Figure 5b, before treatment). Aggregated α-syn mixtures were then incubated with the cells for 24 h, after which the cells were washed to allow α-syn to be taken up. Following this, the same cell was located, and multiple individual mitochondria were extracted from the cell again using the nanotweezer (Figure 5b, after treatment). This provided populations of individual mitochondria from single cells at two time points. The expression of specific mitochondrially encoded genes from each mitochondrion was then analyzed using the method shown in Figure 3a. Overall changes in MT-ND1, MT-ATP6, MT-CO1, and MT-RNR1 expression in the same cells were assessed to provide relative, longitudinal same-cell analyses that are less sensitive to between-cell variability. This was compared with mitochondria derived from corresponding control cells, in which a buffer containing no protein was used instead of the α-syn mixture.

**Figure 5:**
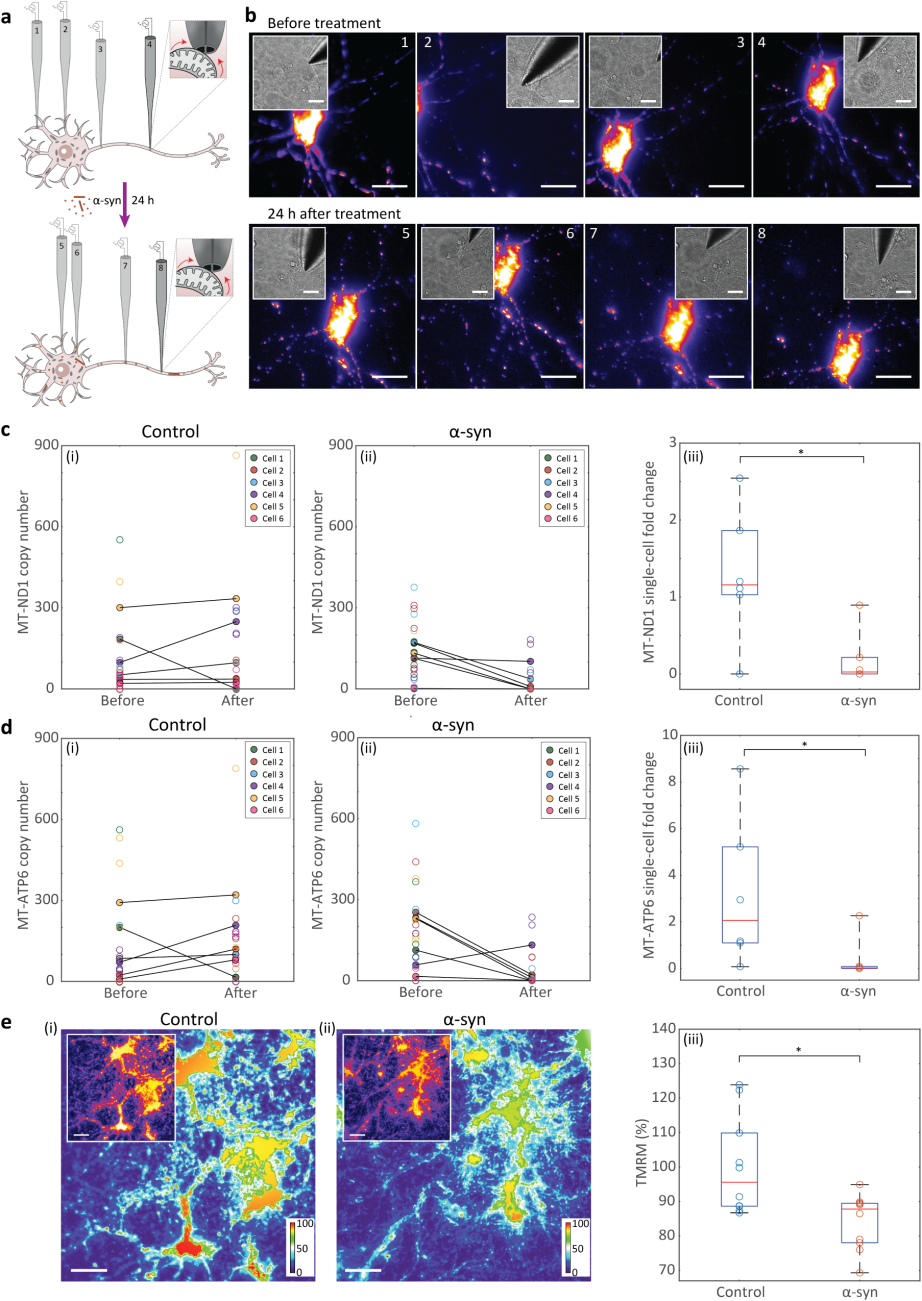
Single-cell tracking of the expression of specific mitochondrially encoded genes with incorporation of aggregated α-synuclein. a) Schematic of same-cell single mitochondrial biopsies with application of aggregated α-synuclein. 3–4 single mitochondria were extracted from a DIV 14 neuron, 0.5 μM aggregated α-synuclein was incubated with the cells for 24 h, the cells were then washed, and a further 3–4 single mitochondria were extracted from the same cell. b) Representative fluorescent images of 8 nanobiopsies taken from the same cell. Inset: bright-field images of the nanotweezer inserted into the cell at the targeted mitochondrion. Scale bars = 20 μm. c) Expression of MT-ND1 tracked in control cells (i), expression of MT-ND1 tracked in α-synuclein-treated neurons (ii), and (iii) the fold change of MT-ND1 measured in the same cells in control and α-synuclein-treated neurons (*n* = 6 cells). d) Expression of MT-ATP6 tracked in control cells (i), expression of MT-ATP6 tracked in α-synuclein-treated neurons (ii), and (iii) the fold change of MT-ATP6 measured in the same single cells in control and α-synuclein-treated neurons (*n* = 6 cells). Statistical analysis was performed using a Mann–Whitney nonparametric test (*P < 0.05, ^n.s^ *P* = not significant). Each filled circle in the tracked plots represents the mean copy number of each cell; the unfilled circles represent the individual copy numbers from each mitochondrion, and the black lines link the mean copy numbers in the same cell before and after treatment. Each unfilled circle in the fold change plots represents the fold change in mitochondrial copy number between the same cell’s pre- and post-treatment mean mitochondrial copy numbers. e) Heat map of TMRM-loaded control cells (i), heat map of TMRM-loaded cells following 24 h treatment with aggregated α-synuclein (ii), and (iii) normalized TMRM fluorescence of control and aggregated α-synuclein-treated cells measured as a percentage of the mean normalized fluorescence of control cells (*n* = 10). TMRM fluorescence was normalized to the cell area in each image. Color bars = fluorescent values of the image (arbitrary units). Inset: Corresponding fluorescent images of the TMRM-loaded cells. Scale bars = 20 μm. Statistical analysis was performed using a Mann–Whitney nonparametric test (*P < 0.05, ^n.s^ *P* = not significant). Summary statistics for boxplots: center = median; bounds of box = IQR 25th and 75th percentile; whiskers = minimum and maximum within 1.5 IQR.

Notably, the expression of MT-ND1 and MT-ATP6 genes (which encode for subunits of mitochondrial complexes I and V, respectively), but not MT-CO1 or MT-RNR1 (Figure S5), was significantly lower following α-syn treatment than in the control condition. Our live-cell tracking, therefore, aligns with previous work showing an impact on mitochondrial complexes I and V with the addition of aggregated α-syn or in Parkinson’s disease tissue.^34,35,40^ To understand if the change in specific mitochondrially encoded expression led to a functional impact on the mitochondria, a TMRM assay was performed on α-syn-treated and control cells (Figure 5e). This showed a significant decrease in MMP in α-syn-treated cells compared to control cells.

These results suggest that the presence of aggregated α-syn decreases the expression of the mitochondrially encoded genes MT-ND1 and MT-ATP6 in neurons, and this is concurrent with a decrease in MMP. However, we did not find a relationship between the proximity of aggregates to mitochondria and the effect on gene expression. It is possible that the impact on mitochondrial expression becomes more diffuse over time due to mitochondrial motility. Alternatively, the effects of α-syn aggregates on mitochondrial gene expression may not be due to a direct interaction of the oligomers with the mitochondria. Rather, the effect on mitochondria may be indirect, for example, as a downstream effect of α-syn toxicity in other regions of the cell. For example, aggregates may disrupt communication between the mitochondria and the endoplasmic reticulum (ER), thereby affecting lipid exchange and calcium signaling.^41^ They may also trigger an ER-unfolded protein response, which can relay apoptotic cues to mitochondria, which can in turn alter mitochondrial gene expression.^42^ Additionally, the aggregates may alter intracellular transport within neurons by destabilizing microtubules^43^ and/or interfering with motor/adaptor proteins such as Miro.^44^ This can impact mitophagy, which would alter mitochondrial gene expression.

In this work, we used a precise minimally invasive single-cell nanobiopsy technique to extract individual mitochondria from live neurons with high spatial and temporal resolution. Our method maintains cells in their native microenvironments and does not rely on cytoplasmic aspiration or mechanical sampling of cellular contents. We demonstrate that single mitochondria can be extracted from neurons using the nanotweezer without affecting the neuronal viability. The maintenance of MMP following mitochondrial extraction highlights the technique's potential for mitochondrial transplantation, as has been achieved with other single-cell sampling approaches.^18,19^

In practice, specificity in our experiments is achieved through a combination of fluorescence-guided targeting, spatially restricted sampling, and downstream molecular validation. At the sampling stage, mitochondria are fluorescently labeled in live neurons, which allows the nanotweezer tip to be positioned directly adjacent to a visually identified individual mitochondrion before DEP is applied. This greatly reduces the probability of trapping unrelated material. In addition, sampling is performed from morphologically isolated mitochondria in neurites rather than more crowded perinuclear regions, further reducing the ambiguity in target selection. During extraction, successful trapping is confirmed visually by the movement of the selected fluorescent mitochondrion toward the nanotweezer tip and the loss of the signal from its original position. At the analysis stage, specificity is further supported by the molecular workflow. For mtDNA analysis, mitochondrial targets are amplified, and appropriate controls are included to exclude contamination or nonspecific carryover. For mtRNA analysis, we include a target-enrichment purification step using surface-functionalized magnetic beads to isolate mitochondrial transcripts while minimizing mtDNA carryover. Thus, although the DEP trapping mechanism is not chemically selective, the overall workflow ensures that the reported signals derive from the targeted mitochondria. The specificity of the workflow may be further enhanced in the future using affinity-assisted or surface-functionalized trapping strategies.

Our development of methods for single-mitochondrial analysis demonstrates that information about mtDNA copy number, mtDNA sequences from which mtDNA mutations can be identified, and expression of a set of mitochondrially encoded genes can be obtained from a single mitochondrion. Our attempts at direct amplification-free nanopore sequencing were unsuccessful, most likely because a single mitochondrion contains very few mtDNA copies, which may inevitably get lost during sample and library preparation. We show that it is possible to analyze a selected set of mitochondrially encoded transcripts from a single mitochondrion. While the principal advance of this work is the development and demonstration of a platform for live-cell single-mitochondrion tracking and analysis, it may open opportunities for highly localized mitochondrial gene expression tracking and linking single-organelle molecular changes with cellular function in the future. We further demonstrate the feasibility of repeated sampling from the same live neuron and detection of treatment-associated directional changes in selected mitochondrial transcripts.

By measuring specific mitochondrial transcripts in the same cells over time, we tracked selected transcript responses to the presence of α-syn aggregates in single neurons. While only preliminary and not reflecting the full chronic biology of endogenous α-syn accumulation in neurodegeneration, such as Parkinson’s disease, we revealed that these aggregates can lead to a downregulation of genes encoding subunits of mitochondrial complexes I and V in an acute toxicity model. Given the dynamic nature of gene expression within cells, our approach shows a proof of concept for tracking targeted mitochondrial transcripts of a cell from its ground state to its treated state, with the potential to unmask the inherent heterogeneity and variability of cells and provide a more accurate depiction of single-cell responses. Interestingly, we found no correlation between the downregulation of MT-ND1 and MT-ATP6 at the single-cell level and the proximity of α-syn oligomers to individual mitochondria. With further sampling and analysis, this may suggest that mitochondrial dysfunction may result from interactions with α-syn species that precede aggregate formation.

Overall, we demonstrate a powerful tool for extracting and analyzing individual mitochondria in living neurons. A significant advantage of the method outlined here is the exceptional precision of the nanotweezer for selecting a mitochondrion of interest within a specific region of a cell, combined with minimal impact on the cell, enabling live single-cell measurements. Current limitations of the technique include the number of mitochondrially encoded genes that can be analyzed from a single mitochondrion, which limits comprehensive gene expression analysis of a single organelle, and the analysis of low-abundance transcripts. Improved preservation of nucleic acid content within each mitochondrion, combined with the ongoing development of highly sensitive sequencing methods, will further increase the power of the technique. While we did not find a stable mitochondrially encoded housekeeping gene in our analyses, other normalization metrics may be incorporated in the future, such as mitochondrial volume. Larger studies will also be required to fully establish the extent of mitochondrial heterogeneity and mitochondrial gene-expression responses in single cells. We anticipate that nanotweezers will become a valuable technique for studying the mitochondrial landscape of living cells.

## Methods

### Primary Neuron Culture

Primary hippocampal and cortical neuronal cultures were isolated from E18 Sprague–Dawley rats. Cells were plated at a density of 25,000 cells/cm^2^ on 35 mm glass-bottom dishes (Greiner Bio-One) or gridded 35 mm glass-bottom dishes (Ibidi). Dishes were precoated with 0.25 mg/mL poly-l-lysine hydrobromide (Sigma-Aldrich) overnight. Cells were attached overnight in Minimum Essential Medium (Gibco) supplemented with 10% horse serum (Gibco), 1 mM pyruvic acid (Sigma-Aldrich), and 0.6% glucose solution (Sigma-Aldrich). Cells were then cultured in Neurobasal (Gibco) supplemented with 2% B27 (Gibco), 1% GlutaMAX (Gibco), 0.6% glucose (Sigma-Aldrich), and 1% penicillin-streptomycin (Gibco) at 37 °C with 5% CO_2_. Half-media changes were performed every 3–4 days. Cells were transfected by lipofection 2 days before experiments using Lipofectamine 2000 (Life Technologies) and the fluorescent mitochondrial reporter DsRed2-Mito-7. For TMRM analysis, cells were incubated with 20 nM TMRM (Life Technologies) in cell maintenance media for 30 min at 37 °C and then washed in PBS and replaced with fresh media. DsRed2-Mito-7 was a gift from Michael Davidson (Addgene plasmid #55838; http://n2t.net/addgene:55838; RRID:Addgene_55838).

### α-Synuclein Preparation and Characterization

N-terminally acetylated α-synuclein monomers were expressed and purified using *Escherichia coli* as previously reported.^49^ The concentration was estimated from the absorbance at 275 nm by using a molar extinction coefficient of 5600 M^–1^·cm^–1^. The aggregation conditions were based on an established protocol.^34,45,46^ Briefly, 800 μL of α-syn monomers (70 μM, 25 mM Tris, 100 mM NaCl, pH 7.4) were ultracentrifuged for 1 h at 90,000 rpm and 4 °C to remove insoluble aggregates. The top 600 μL of the supernatant was collected to isolate the monomers, while the remaining volume was discarded. 400 μL of this monomer sample was mixed with 0.01% NaN_3_ to prevent bacterial growth during aggregation. The mixture was then aggregated by incubating at a temperature of 37 °C with shaking at a constant speed of 200 rpm for 24 h.

For the production of fluorescently labeled α-synuclein aggregates, a fluorescently labeled aptamer specific to α-synuclein was used (5′-GGTGCGGGACTAGTGGGTGTGTTTTTT-ATTO647-3′, Integrated DNA Technologies). Aggregated α-synuclein was incubated with the aptamer for 1 h. The mixture was then washed with PBS and filtered 5 times using Amicon Ultra 100 kDa centrifugal filters to remove unbound aptamer and monomer.

α-Synuclein samples were characterized by AFM. α-Synuclein samples were placed on mica sheets and dried with a nitrogen gun. AFM imaging was performed using an Asylum MFP-3D microscope in tapping mode. Nanosensors PPP-FMR tips (res ∼ 75 kHz, nominal tip radius: 7 nm, nominal spring constant: 2 N/m) were used and tuned to a target tapping amplitude of 1 V. Scans were sized at 90 μm^2^, with 256–512 points per line and a scan rate of 0.5 Hz.

### Nanobiopsy

Nanotweezers were fabricated, and single mitochondrial nanobiopsies were performed using the nanotweezer, as reported previously.^25^ Briefly, mtdsRed-transfected neurons were mounted on an inverted optical microscope (IX71 or IX83, Olympus). Copper wires were inserted into each chamber of the nanotweezer, and the opposite ends were connected to a function generator (TG2000, TTi). The nanotweezer was mounted above the cells and manipulated using a micromanipulator (PatchStar, Scientifica). Guided by bright-field illumination and fluorescence microscopy, the nanotweezer was manually inserted into the neurite of a neuron adjacent to a labeled mitochondrion. An AC voltage (1 MHz, 15 V peak-to-peak) was applied using the function generator to trap a single mitochondrion dielectrophoretically. The nanotweezer was then retracted from the neuron while maintaining the DEP force, and the trapped mitochondrion was transferred to a PCR tube by pressing the tip into the tube containing 4 μL lysis buffer (100 mM Tris-HCl [pH 8], 150 mM NaCl, 20 mM EDTA, 0.2% SDS) in nuclease-free water. For mtRNA analysis, the lysis buffer also contained 1 unit of RNase inhibitor (#N8080119, Applied Biosystems).

### Single Mitochondrion Lysis and Purification

1 μL of 0.05 mg Proteinase K was added to the lysis buffer containing the isolated mitochondrion, and the sample was lysed at 55 °C for 30 min. For mtDNA analysis, single mitochondrion lysates were purified using the AMPure XP Bead-based reagent (Beckman Coulter) with a modified protocol. The reagent was vortexed before use to ensure homogeneous bead distribution. 7 μL of beads were added to 5 μL of mitochondrial lysate (1.4×) in a 0.5 mL tube, mixed by gentle pipetting 10 times, and then incubated for 5 min at room temperature to allow the DNA to bind to the beads. The tube was placed on a magnetic rack (#S1509S, NEB) for at least 2 min to allow the beads to separate from contaminants and lysis buffer components. The supernatant was removed, leaving approximately 2 μL in the tube. While still bound to the magnet, the beads were washed twice with 50 μL of fresh 70% ethanol in nuclease-free water. Full removal of the supernatant was performed at each wash step to further remove contaminants and lysis buffer components. Immediately after the last wash step, to minimize bead drying, 5–10 μL of nuclease-free water was added to the beads and mixed thoroughly, away from the magnetic rack. Beads were incubated for 2 min to elute DNA. The tube was then placed back on the magnetic rack for 2 min to ensure the solution was fully transparent, and the entire eluate was transferred to a PCR tube.

For mtRNA analysis, two types of surface-functionalized magnetic beads were generated to allow for specific binding to rat mtRNAs. Dynabeads MyOne Carboxylic Acid (#65011, Invitrogen) 1 μm magnetic beads were functionalized with oligonucleotides complementary to mtRNAs via a carbodiimide coupling reaction following the manufacturer’s protocol. An oligonucleotide comprising a repeated sequence of 25 thymine nucleotides for binding to polyadenylated RNAs (5′-H_2_N–C_6_-PO_4_^–^oligo­(T)_25_-3′, Integrated DNA Technologies), and a second oligonucleotide comprising a sequence complementary to 12S rRNA (5′-H_2_N–C_6_–PO_4_-oligo­(TAATAAGGTTCGTGTGAAAGGTCAT)-3′, Integrated DNA Technologies) were incorporated into the reaction.

### qPCR Primers and Probes

All primers and probes used for qPCR are listed in Table S1. Primers and probes were designed using Primer-BLAST (https://www.ncbi.nlm.nih.gov/tools/primer-blast/) or PrimerQuest (https://eu.idtdna.com/PrimerQuest/) and were ordered commercially (Integrated DNA Technologies). For multiplexed qPCR, primer/probe compatibility was validated using the OligoAnalyzer Tool (https://eu.idtdna.com/pages/tools/oligoanalyzer/) and the Eurofins Genomics Oligo Analysis Tool (https://eurofinsgenomics.eu/en/ecom/tools/oligo-analysis/).

### qPCR

For mtRNA analysis, purified mtRNA samples were converted to cDNA using the iScript cDNA Synthesis Kit (Bio-Rad) following the manufacturer’s instructions. qPCR reactions were performed using a StepOnePlus Real-Time 96-well PCR system (Applied Biosystems) in MicroAmp optical 0.1 mL 96-well plates (Applied Biosystems) or using a QuantStudio 6 Flex system in MicroAmp optical 384-well plates (Applied Biosystems). For initial validation experiments (Figure 2), singleplex reactions were performed on cDNA samples using SsoAdvanced Universal SYBR Green Supermix (Bio-Rad) over 40 cycles, with thermocycling and reaction conditions as per the manufacturer’s protocol. A melt curve stage was run after the amplification protocol by increasing the temperature at a rate of 0.5 °C/s from 60 to 90 °C. All other experiments were performed as duplex reactions using PrimeTime Gene Expression Master Mix (Integrated DNA Technologies). A 2:1 primer:probe ratio (500 nM:250 nM) was incorporated with thermocycling over 40–45 cycles according to the manufacturer’s protocol. Copy numbers for all targets were determined by absolute quantification using a standard curve of known copy numbers. Standard curves were constructed by a dilution series of a positive control for each target. Each positive control was a known amount of the amplicon or a region of DNA comprising the amplicon for the target primers to bind to. Standard curves were run on the same plate where possible. Otherwise, the most recent standard curve was used, and the threshold for sample target amplification was set to the standard curve’s threshold.

### Sequencing

A 558 bp region of the MT-RNR2 gene of mtDNA was amplified using high-fidelity PCR (Q5 High-Fidelity 2× Master Mix, New England Biolabs) according to the manufacturer’s protocol. The PCR product was purified using the GeneJET Gel Extraction and DNA Cleanup Micro Kit (Thermo Scientific), and the concentration was determined using a Qubit Fluorometer (Invitrogen). 150 fmol of target amplified DNA was sequenced using the MinION device (Oxford Nanopore Technologies) with the MinION Flow Cell (R10.4.1) and the Ligation Sequencing Kit V14 (SQK-LSK114) according to the manufacturer’s protocol. The MinKNOW software (Oxford Nanopore Technologies) was used to collect the raw data and convert it into basecalled reads. Post-basecalling analysis was performed using the EPI2ME platform (Metrichor Ltd.) for sequence alignment.

### Analysis

Images were analyzed using Fiji. Background subtraction was performed on all images. For TMRM quantification, background subtraction was applied uniformly across control and α-syn-treated conditions. Cell areas were defined using Otsu’s thresholding segmentation. TMRM fluorescence was then normalized to the cell area in each image, and the normalized fluorescence in each condition was expressed relative to the mean normalized fluorescence of the control cells. Sequencing analysis was performed using Integrated Genomics Viewer (IGV) and Samtools.^47,48^ Data processing was performed using MATLAB 2020 or Origin 2020. Normality and statistical tests were performed using GraphPad Prism 8. The Shapiro–Wilk test was used to test for normality before all statistical tests. A nonparametric test was used if the data failed the normality test. *P* < 0.05 was considered statistically significant.

## Supplementary Material



## Notes

Mitochondrial membrane potential measurements during single mitochondrion isolation; standard curve for absolute quantification of mtDNA copy number; AFM characterization of α-synuclein aggregation; MT-ND1, MT-ATP6, MT-CO1, and MT-RNR1 standard curves for absolute transcript quantification; same-cell transcript tracking of MT-CO1 and MT-RNR1 with application of aggregated α-synuclein; table of primer and probe sequences used for qPCR (PDF)
